# Phantom limb perception interferes with motor imagery after unilateral upper-limb amputation

**DOI:** 10.1038/srep21100

**Published:** 2016-02-16

**Authors:** Yuanyuan Lyu, Xiaoli Guo, Robin Bekrater-Bodmann, Herta Flor, Shanbao Tong

**Affiliations:** 1 School of Biomedical Engineering, Shanghai Jiao Tong University, Shanghai 200240, China; 2 Department of Cognitive and Clinical Neuroscience, Central Institute of Mental Health, Medical Faculty Mannheim, Heidelberg University, Mannheim 68169, Germany

## Abstract

A potential contributor to impaired motor imagery in amputees is an alteration of the body schema as a result of the presence of a phantom limb. However, the nature of the relationship between motor imagery and phantom experiences remains unknown. In this study, the influence of phantom limb perception on motor imagery was investigated using a hand mental rotation task by means of behavioral and electrophysiological measures. Compared with healthy controls, significantly prolonged response time for both the intact and missing hand were observed specifically in amputees who perceived a phantom limb during the task but not in amputees without phantom limb perception. Event-related desynchronization of EEG in the beta band (beta-ERD) in central and parietal areas showed an angular disparity specifically in amputees with phantom limb perception, with its source localized in the right inferior parietal lobule. The response time as well as the beta-ERD values were significantly positively correlated with phantom vividness. Our results suggest that phantom limb perception during the task is an important interferential factor for motor imagery after amputation and the interference might be related to a change of the body representation resulting from an unnatural posture of the phantom limb.

Motor imagery has been used for the treatment of phantom limb pain (PLP)^1^, motor rehabilitation^2^, and the development of brain-computer interfaces for controlling a prosthesis^3^. Many researches have focused on motor imagery after amputation and the evidence of whether the capability for motor imagery in amputees is impaired remains inconsistent^4^,^5^,^6^. Potential factors contributing to motor imagery impairment after amputation, such as phantom limb perception, were suggested but still need to be systematically investigated. Nico *et al.*^4^ speculated that the change in body schema produced by the amputation and the phantom limb might be a possible cause of increased difficulty in motor imagery. Curtze *et al.*^5^ also proposed that phantom sensation during testing has an effect on motor imagery; however, only two amputees in their study reported phantom sensation during testing. Until now, there is no direct and reliable evidence showing a relationship between the performance of motor imagery and phantom experiences in amputees.

Although the phantom limb is perceived to be an integral part of the body^7^, it is different from an intact limb with a distinct shape, position or posture, leading to an unusual body schema^8^. Patients often state that the phantom occupies a ‘habitual’ posture^9^, sometimes awkward, like telescoped with the limb drawn up into the stump or extended over the normal length^10^. Some amputees describe their phantom limb as being incomplete^11^, and in some cases, the phantom limb is completely paralyzed^12^.

Several studies have demonstrated that the body schema influences motor imagery. Usually, the authors used a hand mental rotation task which involves motor imagery with mental simulations of movements of one’s own hand^13^. Unnatural adopted postures (e.g., keeping one arm flexed^14^, or holding one arm behind their back^15^, or with hands in close contact with each other^16^) or faulty proprioceptive input^17^ interfered with the mental rotation task. Patients with neglect or apraxia, which involve a disorder of the body schema, showed a deficit in mental rotation performance^18^,^19^. In transcranial magnetic stimulation (TMS) studies on motor imagery, lower levels of corticospinal excitability were found with incompatible postures when either the physical posture^20^ or an imagined posture^21^ was manipulated. These findings all imply that motor imagery of body parts and corresponding brain activity should be affected when the body schema is disrupted by amputation or by a distorted phantom limb.

In the present study, the influence of phantom limb perception on motor imagery was investigated by comparing upper-limb amputees with or without perception of a phantom limb with healthy controls using a hand mental rotation task. The response time (RT), which rises monotonically with the increased rotation angle^22^, estimates the ability of participants to mentally rotate their own hands towards the stimulus orientation. Event-related (de)synchronization (ERD/ERS), which reflects the cortical activation^23^, is also related to mental rotation ability^24^. By combining behavioral and ERD/ERS measures, we tested whether phantom limb perception interferes with the motor imagery of amputees. Furthermore, because of the limited spatial information provided by traditional ERD/ERS, source localization was used to determine which brain areas are involved in processing the interference.

## Results

### Behavioral Results

Three amputees (A2, A8 and A26) experienced PLP in their daily life, and only A26 reported PLP during the task. Seven amputees felt residual limb pain (RLP) in daily life, but none of them had RLP during the task. Twenty-one amputees perceived a phantom limb in their daily life, and fifteen of them reported a vivid phantom limb perception during the task. Six amputees with daily phantom limb perception did not perceive a phantom limb during the task; however, three amputees (A14, A22 and A24) who had never perceived a phantom limb in the last year, reported a phantom limb perception during the task.

**Figure 1:**
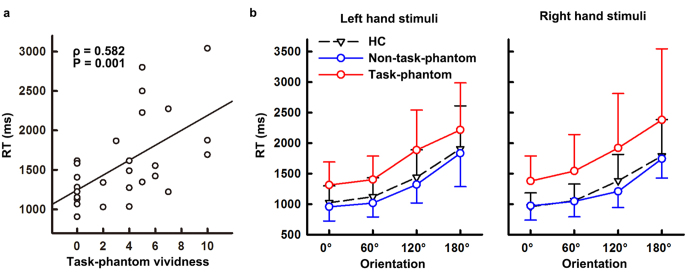
Response time of mental rotation task, showing an interference by phantom limb perception during the task. (**a**) Spearman correlation between response time (RT) and task-phantom vividness. (**b**) Response time (RT) to left hand stimuli and right hand stimuli of healthy controls (HC), amputees with a task-phantom (task-phantom) and amputees without a task-phantom (non-task-phantom). Error bars indicate standard deviation of the mean.

All the healthy controls (HC) and amputees successfully completed the task. The accuracy rates of both controls and amputees exceeded 94.5% and did not show a significant difference between both groups [HC: 96.2 ± 6.6%, amputees: 94.8 ± 9.6%, F(1,52) = 1.093, P = 0.186]. Spearman correlation analyses showed that the overall RTs were significantly positively correlated with the phantom vividness during the task (ρ = 0.582, P = 0.001, two-tailed, Fig. 1a), and so were the RTs for either left or right hand stimuli (left hand stimuli: ρ = 0.557, P = 0.003; right hand stimuli: ρ = 0.540, P = 0.004). However, RTs were not significantly correlated with phantom sensation in daily life from neither intensity (ρ = −0.119, P = 0.554), nor frequency (ρ = −0.107, P = 0.594) or intensity/frequency (ρ = −0.077, P = 0.701), indicating the important role of task-related phantom limb perception for mental rotation performance. Therefore, the amputees were divided into two subgroups according to whether or not they perceived a phantom limb during the mental rotation task, i.e., amputees with a task-phantom (denoted as task-phantom group) and without a task-phantom (denoted as non-task-phantom group).

The repeated measures analysis of variance (ANOVA) on RTs showed a significant main effect of ORIENTATION [F(3,153) = 74.904, P < 0.001, Fig. 1b], which is referred to as an angular disparity, i.e., RTs increased monotonically with orientation from upright (0°) [RT of 0°: 1105.65 ± 331.84 ms, 60°: 1207.51 ± 397.77 ms, 120°: 1550.96 ± 587.62 ms, 180°: 1988.35 ± 743.90 ms, pairwise comparisons: all |T(53)| ≥ 5.026, P < 0.001, Bonferroni-corrected]. The GROUP effect on RTs was significant [F(2,51) = 6.245, P = 0.004]. Amputees with a task-phantom took more time to recognize the various hands than controls (task-phantom: 1754.80 ± 788.65 ms, HC: 1335.66 ± 547.81 ms, *post-hoc*: P = 0.007); however, RTs of amputees without a task-phantom were comparable to those of control subjects (non-task-phantom: 1262.12 ± 439.37 ms, HC: 1335.66 ± 547.81 ms, *post-hoc*: P = 1.000). No significant main effect of HAND on RTs was observed; however, the HAND × GROUP interaction was significant [F(2,51) = 3.267, P = 0.046]. *Post hoc* tests showed that the preference for left and right hand stimuli was significantly different between controls and amputees with and without a task-phantom. Controls responded significantly faster to right hand stimuli [left hand stimuli: 1373.61 ± 572.98 ms, right hand stimuli: 1297.70 ± 515.98 ms, F(1,26) = 4.816, P = 0.037], reflecting an advantage for recognizing the dominant hand. In amputees without a task-phantom, the advantage to recognize the dominant hand was not significant [left hand stimuli: 1281.63 ± 479.97 ms, right hand stimuli: 1242.61 ± 393.66 ms, F(1,8) = 0.352, P = 0.569]. In amputees with a task-phantom, RTs for either the left or right hand stimuli were prolonged and they also did not show the handedness advantage [left hand stimuli: 1704.18 ± 673.06 ms, right hand stimuli: 1805.42 ± 891.34 ms, F(1,17) = 2.009, P = 0. 174].

The revised Movement Imagery Questionnaire (MIQ-R) score also suggested that motor imagery ability was impaired in amputees with a task-phantom. The score of amputees without a task-phantom was close to ceiling level, with high scores to almost all items (mean ± s.d.: 6.91 ± 0.17). However, the MIQ-R score of amputees with a task-phantom (6.62 ± 0.47) was significantly decreased [non-task-phantom vs. task-phantom: T(22.3) = 2.234, P = 0.036, Cohen’s d = 0.72].

### Scalp Beta-ERD Result

**Figure 2:**
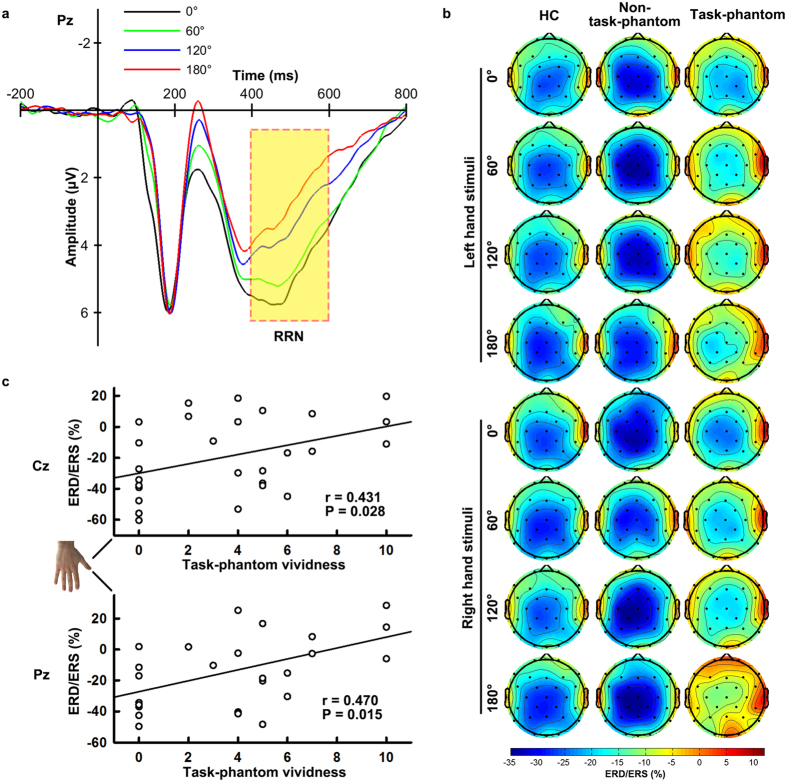
Event-related (de)synchronization (ERD/ERS) of EEG in the beta band in the rotation-related time window. (**a**) Grand-averaged event-related potentials at the Pz electrode for different orientations. The rotation-related time window (400–600 ms) with significant rotation-related negativity (RRN) is highlighted with a yellow background. (**b**) Scalp maps of ERD/ERS in the beta band at 400–600 ms in healthy controls (HC), amputees with a task-phantom (task-phantom) and amputees without a task-phantom (non-task-phantom). (**c**) Partial correlations controlled for RT between task-phantom vividness and ERD/ERS values at Cz and Pz electrodes when amputees mentally rotated the inverted right hand. Hand stimulus was reproduced from ref. 31, with permission.

The rotation-related negativity (RRN), a component that is superimposed on the P300 and increases in amplitude with orientation, was found during 400–600 ms (Fig. 2a). Within the time window for RRN, significant beta-ERD was observed in the frontocentral, central and parietal areas (Fig. 2b). An ANOVA of beta-ERD values at C3, Cz, C4, P3, Pz and P4 electrodes yielded a significant main effect of LATERALITY [F(2,102) = 11.556, P < 0.01]. The beta-ERD was most pronounced in the middle sites (left: −22.54 ± 2.90%, middle: −24.37 ± 2.90%, right: −20.06 ± 2.92%). The interaction REGION × LATERALITY [F(2,102) = 4.410, P = 0.015] was also significant, as shown by more pronounced middle central beta-ERD than that in the middle parietal area [Cz: −25.30 ± 2.88%, Pz: −23.44 ± 2.98%, F(1,51) = 4.617, P = 0.036]. Overall, both controls and amputees without a task-phantom exhibited stronger beta-ERDs than amputees with a task-phantom, although the GROUP effect was insignificant [HC: −23.83 ± 3.66%, non-task-phantom: −27.57 ± 6.34%, task-phantom: −15.58 ± 4.48%, F(2,51) = 1.532, P = 0.126]. The interaction ORIENTATION × GROUP on beta-ERD was significant [F(6,153) = 4.214, P = 0.002]. In controls and amputees without a task-phantom, the beta-ERDs for different orientations were comparable [ORIENTATION effect in HC: F(3,78) = 1.635, P = 0.188 and non-task-phantom: F(3,24) = 0.449, P = 0.720]; whereas in amputees with a task-phantom, a significant ORIENTATION effect was observed [F(3,51) = 6.278, P = 0.004] as the beta-ERD weakened with the orientation (0^o^: −19.72 ± 4.80%, 60^o^: −16.34 ± 4.31%, 120^o^: −14.84 ± 4.92%, 180^o^: −11.42 ± 6.01%). As a result, the disparity among the three groups increased with the orientation, and almost reached a significant level at the inverted (180^o^) orientation [HC: −25.32 ± 4.07%, non-task-phantom: −27.77 ± 7.06%, task-phantom:−11.42 ± 4.99%, F(2,51) = 2.864, P = 0.066]. Significant positive correlations were observed between task-phantom vividness and the beta-ERD values. Generally, larger ERD values correspond to reduced ERD phenomena. A more vivid phantom was associated with less pronounced beta-ERD at the middle central and parietal lobes when amputees mentally rotated the inverted (180^o^) missing (right) hand (partial correlation controlled for RTs, Cz: r = 0.431, P = 0.028; Pz: r = 0.470, P = 0.015, two-tailed, Fig. 2c).

### Source Beta-ERD Result

**Figure 3:**
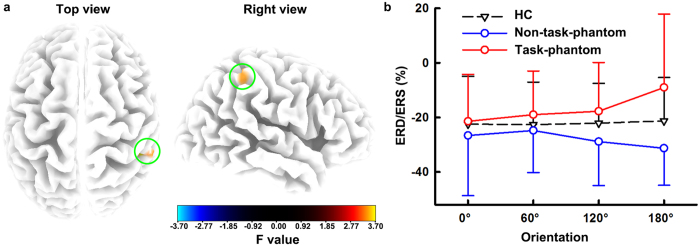
Source localization of the angular disparity of event-related (de)synchronization (ERD/ERS). (**a**) Statistical parametric maps of the ORIENTATION × GROUP interaction for ERD/ERS of each voxel. The green circles highlight the region with significant ORIENTATION × GROUP interaction. (**b**) Averaged ERD/ERS of eleven voxels in the significant cluster of healthy controls (HC), amputees with a task-phantom (task-phantom) and amputees without a task-phantom (non-task-phantom) with respect to different orientations. Error bars indicate standard deviation of the mean.

The beta-ERD source analysis showed a cluster in the right parietal lobe with a significant ORIENTATION × GROUP interaction (Fig. 3a). The cluster contained eleven voxels [maximal at 50, −40, 50; F(6, 153) = 3.695, P = 0.005], including nine voxels in the right inferior parietal lobule (IPL) and two voxels in the right postcentral gyrus (Table 1). Further tests showed that the mean of beta-ERD of eleven voxels in this cluster had similar statistical results as the scalp beta-ERD (Fig. 3b). That is, the beta-ERD of the cluster was comparable for different orientations in controls [F(3,78) = 0.223, P = 0.880] and amputees without a task-phantom [F(3,24) = 2.244, P = 0.109], whereas it weakened with the orientation in amputees with a task-phantom [0^o^: −21.42 ± 17.16%, 60^o^: −18.97 ± 15.97%, 120^o^: −17.75 ± 17.75%, 180^o^: −9.03 ± 26.93%, F(3,51) = 4.806, P = 0.013]. At the inverted (180^o^) orientation, the beta-ERD of the cluster was significantly different in the three groups [HC: −21.27 ± 15.88%, non-task-phantom: −31.28 ± 13.58%, task-phantom:−9.03 ± 26.93%, F(2,51) = 4.675, P = 0.014]. Pairwise comparisons with Bonferroni correction showed that amputees with a task-phantom exhibited a significantly reduced beta-ERD compared with amputees without a task-phantom (P = 0.016). However, no significant correlations were observed between the beta-ERD values and task-phantom vividness in this cluster (P ≥ 0.216).

## Discussion

In this study, the effect of phantom limb perception on motor imagery was investigated in unilateral upper-limb amputees using a hand mental rotation task. Amputees with a task-phantom exhibited prolonged response times and reduced rotation-related neural activity although the motor imagery ability of amputees without a task-phantom was comparable to that of healthy controls. Task-phantom vividness was significantly positively correlated with response time and the rotation-related beta-ERD. Our results suggest that phantom limb perception is an important interference factor in motor imagery after amputation.

In amputees without a task-phantom, behavioral performance and brain activity were equivalent to that for healthy controls, indicating that their ability of motor imagery was unaffected even many years after amputation. This implies that peripheral modifications induced by unilateral limb loss do not prevent and/or affect intrinsic motor imagery when amputees are free from a task-phantom. One possible explanation is that amputees retain the motor pathways for simulation and complete motor imagery through motor memory. Although motor imagery generally parallels the corresponding real action, it is more associated with the early stage of motor control (i.e., motor planning/preparation), with reduced involvement of end-stage movement execution-related processes^25^,^26^. Different brain activations between imagined and actual movements were also observed in amputees^27^,^28^. Execution of phantom limb movements activated the primary somatosensory cortex, the primary motor cortex and the anterior lobe of the cerebellum, while imagination activated the parietal and occipital lobes, and the posterior lobe of the cerebellum^28^. Even when amputees were unable to voluntarily move the phantom, their corresponding movement representations were still intact in the motor area^29^. After amputation, it is possible that loss of overt motor output to the amputated body specifically affects the end-stage movement execution-related processes but not the early-stage planning or motor imagery. One compelling demonstration is that amputation reduced the speed of voluntary movements with the phantom limb but did not change the speed of imagined movements^6^. Similarly, lower-limb amputation did not affect the performance of the mental rotation of feet^5^. However, another study reported less accuracy and slower speed of mental rotation of hands in upper-limb amputees^4^. Such controversial results might be due to the different incidence of phantom phenomena during the task (e.g., 75% in^4^ and 13% in^5^).

Although motor imagery was not disabled by unilateral limb loss in amputees with a task-phantom, their speed was significantly reduced. One possibility is that unnatural postures of the phantom limb interfere with motor imagery of the corresponding body part. As a representative instance of motor imagery, the hand mental rotation task requires the participants to mentally rotate their own hand to match the hand presented in the experiment^30^. This process has been shown to be involuntarily influenced by the physical configuration of the participants’ real hand^15^,^31^. People spontaneously imagine a spatial transformation of their hand from its current orientation rather than from a fixed canonical one^32^. When their hands are in an unnatural posture, for example, hands with intertwined fingers and behind the back, their judgment is slowed down^31^. Detailed analyses found that the body-part posture effect on the mental rotation was specific for the dominant side of right-handers^15^,^33^. In our study, all amputees were right-handed before amputation and had their dominant hand amputated. The vivid unnatural proprioceptive input of their phantom hand, which was their dominant hand before, might cause interference with the mental rotation of the corresponding hand. However, phantom limb perception did not appear to selectively interfere with the judgment for the missing (right) hand but also the intact (left) hand. We speculate that amputees who lost their right dominant hand still keep the strategy of mental rotation of right-handers, i.e., they might prefer to mentally rotate their dominant hand when a rotated non-dominant hand is presented, and thus making a decision based on shape-matching^34^. Similarly, a non-lateralised effect of unilateral amputation was found when subjects were required to perceive visual content in action-relevant objects^35^. The interference by phantom limb perception might also be associated with attention. As attention is involved in body awareness^36^, amputees with a task-phantom need to allocate additional attentional resources to perceive the phantom limb and therefore might be distracted away from the mental rotation task. Similarly, a distraction effect of PLP was assumed when amputees performed a visual oddball task^37^. The P300 component, which is associated with attention, was significantly stronger in the PLP-patients, and the P300 amplitude was positively correlated with the intensity of PLP^37^. In the present study, the interference with motor imagery by phantom limb perception was also demonstrated by the correlation between the response time and task-phantom vividness. That is, a larger interference arose from a more vivid phantom and led to a poorer behavioral performance.

Reduced beta-ERD in the rotation-related time window of amputees with a task-phantom, especially at large orientations, provides electrophysiological evidence of interference by phantom limb perception. As ERD reflects the oscillatory aspects of cortical activation (e.g., attention^38^,^39^ and motor imagery^40^,^41^), our beta-ERD results suggest a central and parietal hypo-activation in amputees with a task-phantom compared with those without a task-phantom and healthy controls. We speculate that such a cortical hypo-activation in amputees with a task-phantom might arise from the unnatural posture of the phantom hand since a previous study using TMS had demonstrated that incompatible postural signals could lead to a smaller motor evoked potential area and a lower increase in corticospinal excitability than compatible ones^20^. Further, the beta-ERD decreased with orientation in amputees with a task-phantom, which might be due to an increasing incongruence between amputees’ phantom hand and the stimulus hand with the stimulus hand rotating from upright to inverted ones. The most prominent interference by phantom limb perception occurred when subjects mentally rotated the missing hand at the inverted orientation. In this case, the magnitude of the middle central and parietal beta-ERD was significantly and positively correlated with task-phantom vividness. The cortical source of this angular disparity effect was mainly located in the right IPL. Amputees with a task-phantom exhibited significantly reduced beta-ERD compared to those without a task-phantom in the right IPL when responding to hand pictures at the inverted orientation. The right IPL was reported to be related to own-body perception and the implementation of the body schema^42^,^43^. This region is specifically involved in the mental rotation of body parts but not for alphabetic characters^44^. Activation in this area was also observed during the perception of distorted body images^45^,^46^. We suggest that the right IPL hypoactivation in amputees with a task-phantom might be related to their rather inflexible, probably distorted body schema.

Phantom limb perception during the task but not that in daily life interferes with motor imagery. Interestingly, phantom limb perception in the test situation did not show a direct correlation with daily phantom experience. Some amputees reported not to feel a phantom limb during the mental rotation task although they usually did. In contrast, the mental rotation task could also elicit a phantom limb perception in some amputees who had not perceived a phantom limb in the last year before the test. Previous studies have also reported the possibility of reappearance of a phantom limb after its complete disappearance by stimulation or under intense concentration^9^,^47^. In the present study, the occurrence of a phantom limb in the task may be related to the kinesthetic nature of mental rotation task^48^.

We speculate that the unnatural posture of the phantom limb might be a possible cause of the interference with motor imagery. Although the corresponding posture of the phantom limb was not assessed in the experiment, we re-interviewed the participants via telephone after the experiment. These postural data confirmed that the phantom limb was often occupied by an unnatural habitual posture, such as a clenched fist (in n = 14), a telescoping distortion (in n = 4) or rather volatile postures (in n = 7), which is in line with the previous report by Ramachandran & Hirstein^9^. Amputees with a phantom limb also reported the ability to move it voluntarily except for A2, A3 and A8. As motor control over the phantom limb is regarded as a result of motor system reorganization, and thus associated with PLP^49^, we speculate that the incapability to move the phantom in three subjects might be due to their experience of PLP, since two of them were suffering from PLP, and the third one (A3) reported a history of PLP in the past.

The prolonged mental rotation in amputees with a task-phantom may be a complicated process. The altered beta-ERD within the rotation-related time window was an electrophysiological evidence of interference with the motor imagery process. We further performed a new supplementary control experiment which only included upright back-view hand stimuli to exclude influences other than the motor-imagery sub-stage, i.e. the early visual perception and the later decision making. Seven amputees (including five amputees from the task-phantom group and two newly recruited amputees with a task-phantom) and seven control subjects participated in the new control experiment. There was no significant difference in RTs between amputees and controls (F(1, 12) = 0.791, P = 0.391, RT of controls: 1598.83 ± 431.89 ms, RT of amputees: 1315.74 ± 723.06 ms), suggesting that the prolonged RT of the mental rotation task in amputees with a task-phantom should primarily involve the impaired motor imagery.

In this study, only dominant limb amputees were included. The amputation side is an important factor for motor imagery after amputation. Nico *et al.*^4^ demonstrated that dominant limb amputees suffered more impairments in motor imagery than those who had lost their non-dominant limb. Unnatural postures of the non-dominant hand do not influence the motor imagery in healthy people^15^,^33^. Therefore, the phantom limb perception in non-dominant limb amputees might be differently related to motor imagery and is worth further investigating. Another limitation is the unequal sample size since only a small proportion of the amputees (9 of 27) did not perceive a phantom limb during the mental rotation task. However, this study provides evidence on the interference of a perceived phantom limb with motor imagery. Our results suggest that phantom experiences should be taken into consideration when studying motor imagery in amputees.

## Materials and Methods

### Participants

Twenty-seven right-sided upper-limb amputees (age: 48.48 ± 9.33 years; education: 9.70 ± 2.63 years; sex: 21M/6F) and twenty-seven age- and education-matched healthy controls (age: 48.26 ± 10.05 years; education: 10.85 ± 3.03 years; sex: 20M/7F) took part in this study. According to a Chinese version of a standardized handedness inventory^50^, the participants were all right-handed (for amputees, they were right-handed before amputation). All subjects had normal or corrected-to-normal vision, and reported no difficulty in foot movement and no history of neurological or mental disorders. As phantom limb perception was hypothesized to constitute an important influential factor for motor imagery, the amputees were divided into two subgroups: a task-phantom group (n = 18, age: 48.00 ± 10.54 years; education: 9.50 ± 2.57 years; sex: 14M/4F) and a non-task-phantom group (n = 9, age: 49.44 ± 6.69 years; education: 10.11 ± 2.85 years; sex: 7M/2F). The three groups (HC, task-phantom and non-task-phantom) did not differ significantly in age [F(2,53) = 0.069, P = 0.993] or years of education [F(2,53) = 1.226, P = 0.302]. Independent t-tests showed that the two subgroups of amputees had no significant difference in amputation level, amputation age or the time since amputation (all T(25) <0.973, P ≥ 0.34). The detailed demographic and amputation-related information of amputees is summarized in Table 2. Each subject signed a written informed consent after the nature of the study had been explained to him or her. The experimental protocols were in compliance with the Declaration of Helsinki. This study was approved by the institutional ethics committee of Shanghai Jiao Tong University.

### Behavioral Assessment

Visual and kinesthetic imagery abilities were measured using MIQ-R^51^. Each questionnaire item asked about the ease or difficulty of imagery on a 7-point scale (1: ‘very hard to see/feel’ — 7: ‘very easy to see/feel’). In the items measuring imagery of upper-limb movements, amputees were required to imagine with the intact limb. While assessing full-body movements, both intact and amputated arms were imagined. Two amputees did not complete the MIQ-R inventory as they could not understand the questionnaire. Clinical data related to the amputation as well as PLP, RLP and the phantom vividness in daily life (i.e., daily-PLP, daily-RLP and daily-phantom vividness) were collected before the mental rotation task. Pain magnitude was calculated by dividing the worst pain intensity within the last week or in a typical week involving pain (a numerical rating scale, 0: ‘no pain’ — 10: ‘worst pain imaginable’) by the frequency of pain experienced in the last year (1 — ‘all the time’, 2 — ‘daily’, 3 — ‘weekly’, 4 — ‘several times per month’ and 5 — ‘once or less per month’)^52^. A similar measurement (intensity/frequency) was conducted for daily-phantom vividness and the intensity was measured by a numerical rating scale (0: ‘no vividness’ — 10: ‘extreme vividness’), in response to the question: “How vivid is the feeling of a phantom limb during the last week (or in a typical week)?”^53^. Amputees’ PLP, RLP, and the phantom vividness during the task (i.e., task-PLP, task-RLP and task-phantom vividness) were assessed immediately after the mental rotation task using the above numerical rating scales.

### Hand Mental Rotation Experiment

**Figure 4:**
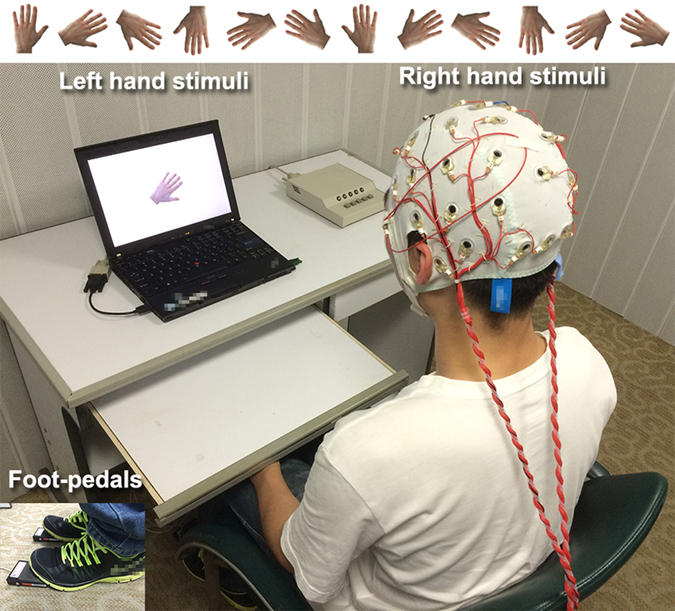
The experimental set-up. Stimuli of left or right hand in back view in six orientations were randomly presented in the screen of a portable computer. The subjects seated in front of the screen, judged whether the picture on the screen is a left or right hand, and responded with foot panels. 32-channel EEG signals were recorded during this task. Hand stimuli were reproduced from ref. 31, with permission.

The subjects were seated in front of a portable computer with their hands (hidden from view) resting and folded on their laps. Amputees wearing a prosthesis were asked to keep their prosthesis on and to place it on their lap during the experiment. Stimulus pictures of the left or right back-view hand (9 cm × 9 cm) at six orientations (0^o^, 60^o^, 120^o^, 180^o^, 240^o^ and 300^o^) were randomly presented on the display (13 inches) (Fig. 4). To induce an explicit motor imagery process^54^, the subjects were asked to imagine rotating their own hand to the orientation of the hand picture, and judge whether the picture was a left or right hand by making a corresponding foot-pedal response, as quickly and accurately as possible. During the experiment, the subjects were required not to move their hands (including the phantom hand). Each experiment consisted of 4 blocks after 1 training block. There was a 3–5 min inter-block break. In each block, there were 96 trials (2 hands × 6 orientations × 8 repetitions). Each trial began with a black fixation cross (800 ms) followed by a randomly selected hand stimulus on a white background. The hand pictures were presented until the participants responded. The presentations of the stimuli were programmed by E-prime software (v2.0, Psychology Software Tools Inc., Pittsburgh, PA). RTs were computed as time elapsed between the appearance of the hand picture and the foot response. Trials with incorrect responses or RTs exceeding the participant’s mean by more than two standard deviations (5.20% of all trials) were excluded in the following analyses.

### EEG Acquisition and ERD/ERS Analysis

The EEG signals were recorded continuously with a 32-channel Ag/AgCl EasyCap^TM^ (Brain Products GmbH, Munich, Germany). Vertical and horizontal electro-oculograms were also recorded for detecting eye-movements and blinks. Electrode impedances were kept below 20 kΩ during the recording. All electrodes were referenced to FCz. The EEG signals were amplified using the BrainAmp MR Plus amplifier, sampled at 1000 Hz and filtered online with a 100 Hz high cut-off filter.

The EEG signals were preprocessed offline with BrainVision Analyzer (v2.0, Brain Products GmbH, Munich, Germany). The data were band-pass filtered into 0.01–40 Hz and re-referenced to the linked mastoids. Ocular artifacts were corrected using a semi-automatic correction procedure based on the algorithm of independent component analysis. Trials with motion artifacts (EEG amplitude value exceeding ±200 μV or gradient value more than 50 μV/ms, 6.52% of all trials) were excluded by semi-automatically detecting.

ERD/ERS in the beta band was calculated in this study. A rotation-related time window (400 ms to 600 ms) was selected for ERD/ERS analysis. In this time window, a parietal slow component, termed RRN, was specified as the brain activity associated with the mental rotation processes^55^.

All artifact-free EEGs were band-pass filtered into the beta band (13–30 Hz). The ERD/ERS values were calculated as the percentage of power change (decrease or increase) relative to the baseline (−200 ms to 0 ms)^56^. Then, the ERD/ERS data were averaged over all trials by stimulus types and smoothed by averaging all the data in the time window to reduce the variance. Beta-ERD values at six representative electrodes in central and parietal areas (i.e., C3, Cz, C4, P3, Pz and P4) were used for statistical analysis.

### Source Localization

To locate the sources for the changes of the ERD/ERS patterns in amputees, the cortical three-dimensional distribution of the current density of beta oscillations was estimated using the Standardized Low Resolution Electromagnetic Tomography (sLORETA) software. sLORETA is a linear minimum norm inverse solution to the EEG localization inverse problem in Montreal Neurological Institute (MNI) space and has been shown to have no localization bias^57^. The sLORETA map/image, which represents the exact magnitude of the estimated current density, is computed for 6239 voxels (5 mm resolution, restricted to the gray matter/hippocampus)^58^. For each voxel, the ERD/ERS was calculated as the percentage decrement/increment of power density within the rotation-related time window (400 ms to 600 ms) compared with the baseline (−200 ms to 0 ms).

### Statistical Analysis

Paired t-test revealed that the symmetric angles (60^o^ vs. 300^o^, 120^o^ vs. 240^o^) did not show significant differences of RTs for any participant (P ≥ 0.261), which was in line with previous findings in healthy people that angular disparity patterns of back-view hands remain symmetric for medially and laterally rotated stimuli^59^,^60^. Therefore, symmetric orientations (60^o^ and 300^o^, 120^o^ and 240^o^) were collapsed to obtain four categories of orientations (i.e., 0^o^, (±)60^o^, (±)120^o^ and 180^o^) in the statistical analysis. A three-way repeated-measures ANOVA was performed on RTs, taking GROUP (three levels: HC, task-phantom and non-task-phantom) as between-subject factor, and ORIENTATION (four levels: 0^o^, 60^o^, 120^o^, and 180^o^) and HAND (two levels: left hand stimuli and right hand stimuli) as within-subject factors. The beta-ERD values at six electrodes (C3, Cz, C4, P3, Pz and P4) were analyzed by a five-way repeated-measures ANOVA, taking REGION (two levels: central and parietal) and LATERALITY (three levels: left, middle and right) as other two within-subject factors. The beta-ERDs for each voxel was analyzed by a 3 × 4 × 2 (GROUP ×ORIENTATION × HAND) ANOVA. To control the alpha inflation due to multiple comparisons from a statistical test at each voxel, we used a cluster-level correction based on a Monte Carlo simulation using the AlphaSim program in the REST toolbox (http://www.restfmri.net/forum/REST) for MATLAB, according to which clusters (≥4 voxels) at a threshold of P < 0.01 (equivalent to cluster-level P_corrected_ <0.05) were specified as statistically significant^61^. F contrasts were constructed to test for which scalp beta-ERD effects were significant.

Greenhouse-Geisser corrections were used where the assumption of sphericity was not appropriate. All *post-hoc* tests were corrected using the Bonferroni adjustment for multiple comparisons. To capture the changed neural processing underlying the behavioural differences, partial correlation analyses, controlling for RTs, were used to evaluate the association between the task-phantom vividness and the beta-ERD values. All data are presented as mean ± s.d.

## Additional Information

**How to cite this article**: Lyu, Y. *et al.* Phantom limb perception interferes with motor imagery after unilateral upper-limb amputation. *Sci. Rep.* **6**, 21100; doi: 10.1038/srep21100 (2016).

## Figures and Tables

**Table 1:** Voxels that showed significant ORIENTATION × GROUP interaction (P < 0.01).

Structure	Hemisphere	Brodmann Area	F Value	MNI Coordinates		
				X	Y	Z
Inferior Parietal Lobule	R	40	3.695	50	−40	50
	R	40	3.542	45	−35	45
	R	40	3.515	45	−40	45
	R	40	3.408	40	−35	40
	R	40	3.394	40	−35	45
	R	40	3.387	55	−40	50
	R	40	3.365	45	−40	50
	R	40	3.282	50	−40	55
	R	40	3.261	40	−40	45
Postcentral Gyrus	R	40	3.343	50	−35	50
	R	2	3.238	35	−30	40

**Table 2:** Clinical and phantom limb characteristics of amputees.

ID	Age (y)	M/F	Education (y)	Amputation location	Time since amputation	Cause for amputation	Prosthesis use (type, frequency)	MQI-R	Daily-PLP	Daily-RLP	Daily-phantom vividness	Task-PLP	Task-RLP	Task-phantom vividness
A1	29	M	9	Below elbow	7m	Accident	Myoelectric, 90%	7	0	0	2.33	0	0	4
A2	42	F	6	Below elbow	9m	Accident	Myoelectric, 90%	6.125	0.4	0	0.6	0	0	5
A3	36	M	15	Below elbow	9y	Accident	Myoelectric, 10%	7	0	0	0.6	0	0	2
A4	53	M	12	Below elbow	14y	Accident	Aesthetic, 50%	7	0	0	4	0	0	0
A5	53	M	16	Below elbow	33y	Accident	No	7	0	0.4	0.4	0	0	0
A6	35	M	15	Below elbow	7y	Trauma	Aesthetic, 90%	6.625	0	0	2	0	0	4
A7	55	M	12	Below elbow	32y	Accident	No	7	0	1	1.5	0	0	6
A8	59	M	9	Below shoulder	29y	Accident	No	7	2.25	2.5	4.5	0	0	0
A9	57	M	6	Shoulder	30y	Accident	No	none	0	0	10	0	0	10
A10	69	M	9	Below shoulder	36y	Accident	No	5.75	0	0	3.5	0	0	7
A11	56	M	6	Shoulder	7y	Accident	No	6	0	0	1.5	0	0	4
A12	44	F	6	Below shoulder	15y	Accident	No	7	0	0	2.67	0	0	0
A13	53	M	9	Below elbow	13y	Accident	No	6.875	0	0	5	0	0	7
A14	52	M	9	Below elbow	31y	Accident	No	7	0	0	0	0	0	2
A15	53	M	12	Below shoulder	37y	Accident	No	none	0	0	5	0	0	0
A16	55	M	12	Below elbow	17y	Accident	No	7	0	0	3	0	0	5
A17	50	F	9	Below elbow	19y	Accident	No	7	0	0	1.33	0	0	5
A18	57	F	9	Below elbow	15y	Accident	No	7	0	0	1.5	0	0	10
A19	38	M	9	Below shoulder	18y	Accident	No	6.25	0	0	4	0	0	4
A20	56	M	9	Below wrist	15y	Accident	No	7	0	1.25	0	0	0	0
A21	54	M	9	Below elbow	29y	Accident	No	6.625	0	0	2	0	0	6
A22	45	M	9	Below shoulder	28y	Accident	No	7	0	2.25	0	0	0	3
A23	41	M	9	Below elbow	17y	Accident	No	6.625	0	2	0	0	0	0
A24	33	F	9	Below shoulder	15y	Accident	No	5.75	0	0.5	0	0	0	5
A25	44	F	9	Below elbow	9y	Accident	Aesthetic,100%	7	0	0	1.67	0	0	0
A26	48	M	9	Below shoulder	2y	Accident	No	6.5	5	0	3	5	0	10
A27	42	M	9	Below elbow	25y	Accident	No	6.625	0	0	0	0	0	0

Daily-PLP and Daily-RLP and Daily-phantom vividness denote phantom limb pain, residual limb pain and the phantom vividness in daily life, respectively. Task-PLP, Task-RLP and Task-phantom vividness denote phantom limb pain, residual limb pain and the phantom vividness during the task, respectively. M = Male; F = Female; y = years; m = months.
